# Perspectives on wolves after their recolonization in Flanders, Belgium

**DOI:** 10.1098/rsos.231931

**Published:** 2025-03-19

**Authors:** Hilde Vervaecke, Thaana Van Dessel, Peter Galbusera, Joachim Mergeay

**Affiliations:** ^1^ Agro-and Biotechnology Research Group, Odisee University of Applied Sciences, Hospitaalstraat 21, Sint-Niklaas 9100, Belgium; ^2^ Antwerp Zoo Centre for Research and Conservation, Royal Zoological Society of Antwerp, Koningin Astridplein 20-26, Antwerp 2018, Belgium; ^3^ Research Institute for Nature and Forest, Havenlaan 88, Brussels 1000, Belgium; ^4^ KU Leuven, Ecology, Evolution and Biodiversity Conservation, Charles Deberiotstraat 32 - Box 2439, Leuven 3000, Belgium

**Keywords:** wolf, survey, perspective, attitude, human-wolf conflict

## Abstract

At the time of the wolf’s (*Canis lupus*) recolonization in Flanders, public perspectives on this species were not well understood. To address this gap, we conducted a survey gathering demographic and contextual data to explore the relationship between these factors and public perspectives on wolves. We defined perspectives as: attitudes towards wolves, perceptions as whether they belong in Belgium, their mode of arrival, and attitudes towards wolf-related conflicts. Using redundancy analysis, we identified key explanatory variables, including hunting, residency, education, age, gender and dog ownership. Although these factors were significantly associated with perspectives on wolves, their explanatory power was limited, except for being a hunter. Notably, hunters generally had negative perspectives on wolves; however, hunters who stated they had negative attitudes towards hunting showed more positive perspectives on wolves. Conversely, non-hunters with positive attitudes towards hunting showed more negative perspectives. Attitudes towards hunting emerged as the strongest explanatory variable and may serve as a useful proxy for researchers studying wolf perspectives. Recognizing the diversity of stakeholder perspectives, particularly attitudes towards hunting, and underlying ethics could enhance the effectiveness of wolf conservation management.

## 1. Introduction

Over the past 30 years, grey wolves (*Canis lupus*) have been recolonizing various regions across Europe, aided by protection instruments such as the Bern Convention (1982) and the EU Habitats Directive (1992) [1–3]. However, habitat fragmentation and connectivity remain significant challenges [4]. Wolves have demonstrated remarkable adaptability, enabling them to thrive even in human-dominated landscapes [3]. Their return often sparks conflicts, especially in densely populated areas [5], leading to conflicts between wolf conservation and certain human activities, such as livestock farming (e.g. Greece or France: [6,7]). The topic of wolves provokes strong reactions among the public, with opinions sharply divided on whether wolves still have a place in Europe’s now highly anthropized landscapes [8,9]. Residents' attitudes towards both repopulating and established wolf populations play a pivotal role in achieving coexistence, defined as a sustainable balance where humans and carnivores adapt to sharing spaces, supported by institutions ensuring survival, acceptance and safety [10]. Therefore, it is crucial to understand public perspectives and the attitudes of various stakeholder groups towards wolves [11].

Perceptions and attitudes towards wolves can be shaped by demographic and personal characteristics. Some studies have identified more negative attitudes among older individuals [10,12], individuals with lower education levels [10], rural residents, people who are less informed about the topic, and those who engage less in nature-related activities [12,13]. Conversely, positive attitudes have been linked to higher education levels [12,13], higher welfare levels and living in urban environments [12–15]. The association with gender varies, with both males and females having the most negative attitudes depending on the study [15–17]. In south-central Scandinavia, public support for wolf conservation is strongest among urban residents, with positive attitudes increasing with greater distance from wolf habitats. This trend is evident when comparing urban and rural areas, as well as rural areas inside and outside wolf territories [18]. Similarly, in Belgium [19] and the Netherlands [20], the general public from relatively rural areas has demonstrated neutral to negative attitudes towards wolves. Furthermore, research indicates that positive attitudes towards large carnivores as part of a region’s natural fauna are often inversely correlated with the time since the species' extinction [12,21]. Compared with regions without wolves or where wolves have recently recolonized after a long absence, people seem to have more negative attitudes in areas where wolves have always existed [10]. Irrespective of residence in regions with or without wolves, individuals who identify with interest groups likely to directly impact or be impacted by wolf populations, such as farmers and ranchers, exhibit lower tolerance towards wolves [22]. Further, wolves often elicit strong emotional reactions, with fear and perceived threats to human livelihoods, pets and safety contributing to more negative views [23]. These concerns can arise from direct experiences, such as wolves preying on livestock or attacking hunting dogs [24], or from indirect experiences, such as knowing someone who has experienced depredation or being influenced by media coverage of wolf-related issues [11,18,21]. The likelihood of wolf attacks on humans is very low, with only a handful of documented cases in recent decades [25]. The potential risks posed by habituated wolves (e.g. [26,27]) can be dealt with by adaptive management (e.g. [28]).

Multiple studies have identified hunting as a significant factor influencing attitudes towards wolves, with the majority of hunters expressing negative attitudes towards the carnivore [10–12,24,29–31]. In the Flemish region, where the first wolf pair settled, both hunters and farmers exhibited negative perceptions of wolves [19]. These attitudes closely align with the respondents' broader worldview on nature. Farmers and hunters generally adhere to a mastery-over-nature (or domination) perspective and have the most negative views on wolves, in contrast to nature managers, who have more positive views, and local residents, who display neutral to positive attitudes [19]. Scientists who embrace a stewardship-of-nature philosophy tend to have more positive perceptions of large carnivores overall [20]. Such core values guide behaviour and decision-making across various contexts, while attitudes are more context-specific and less enduring [32]. Hunters often fear negative experiences, such as losing hunting dogs to wolves [33–35]. Their negative views are rooted in a historical conflict over resources, with concerns that wolves may reduce prey availability, posing an economic risk for professional hunters and limiting opportunities for leisure hunting [36]. However, some studies have shown that hunters can also value the presence of wolves, particularly when they recognize the species' ecological role or appreciate the experience of seeing wolves in the wild [29,37]. A link has been found between attitudes towards wolves and certain beliefs about hunting; for example, those who agree with the statement ‘we should strive for an abundance of wildlife for hunting’ tend to have negative attitudes towards wolves, while those who believe ‘hunting is cruel’ are more likely to have positive attitudes [38]. This suggests that hunting-related beliefs warrant further study in the context of wolf conservation.

Belgium was one of the last mainland European countries where wolves formally re-established and began reproducing [39,40] after an absence of more than a hundred years. The first officially recognized reappearance of wolves in Belgium occurred in 2016 when wolf DNA was identified on a depredated sheep. At the time of our study, three wolves had settled in Belgium, including a female GPS-collared in northern Germany in 2016, who dispersed to Belgium between September 2017 and January 2018 [39,40]. There was one resident pair of wolves in Flanders, occupying a territory within a military domain, nature reserve, and forests. Additionally, a lone female wolf was present in a nature reserve in Wallonia. In 2019, the lactating female in Flanders disappeared, presumably because of poaching [41]. Wolves typically occupy large home ranges in Europe, ranging from 100 to 2000 km² depending on habitat productivity [42]. However, they can adapt to anthropogenic landscapes, and in areas with high prey density, home ranges may be as small as 80 km² [43,44]. Wolves from neighbouring countries frequently traverse Belgium, with DNA analyses and GPS data confirming that wolves have spontaneously recolonized the country [40,45–48]. During post-juvenile dispersal, wolves can migrate hundreds to thousands of kilometres [43], and nearly 200 wolf packs were estimated to exist within a 1000 km radius of Belgian territories at the time of the study [43,47,49].

To gain a deeper understanding of public perspectives on wolves in Flanders, we conducted an online survey aimed at capturing diverse viewpoints. The survey targeted the general public as well as specific stakeholder groups, including members of nature organizations, farmers and hunters. Our primary objective was to identify and analyse the factors shaping public perspectives on wolves, with the ultimate goal of informing conflict mitigation strategies and promoting sustainable coexistence. The survey framework was informed by previous studies, which have identified several explanatory variables associated with attitudes towards wolves, such as gender, age, education level, ownership of livestock or pets, proximity to wolf habitats, and participation in nature- or hunting-related activities (EU: [3,13,16,23,50]). While these variables provide valuable insights, they often exhibit strong intercorrelations, complicating their interpretation. To address this, we employed a redundancy analysis framework, allowing us to partition the explained variance and disentangle the relative contributions of these variables. In this study, we use the term ‘perspective on wolves’ to encompass both attitudes and perceptions. Attitudes are defined as ‘a psychological tendency expressed by evaluating a particular entity with some degree of favour or disfavour’, reflecting a positive or negative evaluation [51]. Perceptions refer to the ways in which individuals interpret and understand information about wolves [52]. Our analysis focused on a set of classic explanatory variables, supplemented with an additional variable: attitude towards hunting. These variables were linked to a series of response variables, including attitudes towards wolves, perceptions of wolves’ role in Belgium, beliefs about their mode of arrival (spontaneous or human-induced), and views on wolf-related conflicts, which frequently feature in public debates in Flanders. Collectively, these response variables formed our operational definition of ‘perspective on wolves’. This study aims to advance our understanding of how these variables shape public perspectives and ultimately to provide actionable insights for managing human-wildlife interactions.

## 2. Material and methods

We conducted an online Qualtrics survey to gather perspectives on wolves in Flanders (in Dutch; see the electronic supplementary material). The survey was open from 17 January 2019 to 3 May 2019. Belgium, particularly Flanders, is one of the most densely populated regions in Europe, with an average of 485 inhabitants km^−^² [53]. Approximately 60% of the Belgian population resides in Flanders, which accounts for about 44% of Belgium’s total surface area [54]. Flanders is dotted with cities and towns interconnected by an extensive road network. At the time of the survey, there were 3.78 million women (51%) and 3.62 million men (49%) in Flanders, 13 000 hunters with a license, 18 000 active farms and 120 000 members of Natuurpunt, the largest nature-related organization (https://statbel.fgov.be/nl).

We recorded the respondent’s age, place of residence, gender, whether they lived in a city, in a village centre or rurally, education level (lower education: 1, secondary education: 2, bachelor: 3, master: 4, PhD: 5), whether they owned a dog (0 = no, 1 = yes), a cat (0 = no, 1 = yes) and livestock (0 = no, 1 = yes), how often they spent time in nature (daily, weekly, monthly, rarely, scored as 365, 52, 12 and 1, respectively), if they engaged actively in hunting (0 = no, 1 = yes) and we asked them to score their attitude towards hunting (very negative: −2, rather negative: −1, neutral: 0, rather positive: 1, very positive: 2). From the respondent’s recorded postal codes, we identified which respondents live in an area with active wolf presence (7.0%). Information on postal codes was then excluded from the dataset to ensure full anonymity. This yielded 10 explanatory variables. Living in a city, urban area or rural area was coded as 10, 00 or 01, respectively, in two separate variables (city versus urban or rural).

We surveyed participants to assess their attitudes towards wolves in Belgium, evaluating whether their attitude was negative or positive. This was scored on a five-point scale: very negative, negative, neutral, rather positive and very positive. Additionally, we examined their perceptions of whether wolves had been released, had recolonized spontaneously, or if they did not know, and whether they believed wolves belonged in Belgium. The latter was also scored on a five-point scale: definitely yes, probably yes, do not know, probably not and definitely not. We also asked participants to evaluate the intensity of conflict between wolves and humans concerning livestock and recreation. This was scored on a five-point scale: very small, small, do not know, large and very large. Lastly, we inquired how many wolves participants believed were present at the time of the survey, as a measure of knowledge on the topic.

All variables except for the estimated number of wolves were treated as response variables in the multivariate data analyses, representing the ‘perspective on wolves’. For analytical purposes, the response variables originally scored on five-point scales were recategorized into three-point scales (see the electronic supplementary material, Excel file, for details on variable recoding). Perspectives on wolves were categorized as ‘negative’, ‘neutral’ or ‘positive’, based on respondents’ attitudes towards wolves, beliefs about whether there is a place for the wolf in Belgium, and perceptions of conflict. Thus, a ‘negative’ perspective is defined as holding a negative attitude towards wolves, believing that wolves have no place in the landscape, that they were introduced by humans, and associating high levels of conflict with human–wolf interactions. Using different scales to assess survey responses may have introduced variability or noise into the data. We conducted a *Z*-test for proportions to evaluate if the observed sample proportions of characteristics of the respondents significantly differed from the expected population proportions, using a significance level of 0.05 for hypothesis testing. We explored the relationships between the two sets of variables—explanatory (independent) variables and response (dependent) variables—through multivariate linear regressions between data matrices. The redundancy analyses (RDA) were performed using the vegan package [55] in R 4.4 [56] using RStudio 2024.04.2 [57]. RDA examines and interprets multivariate data by combining features of multiple regression and principal component analysis. RDA allows us to quantify the importance (or ‘weight’) of each explanatory variable by measuring how much variation in the response variables is explained by that specific explanatory variable. We first created a global RDA model that included all variables and then determined the marginal effects of each explanatory variable per dataset, considering each variable’s effect independently. A forward selection procedure was then applied, according to [58], where conditionally significant variables (*p* < 0.05) were iteratively added to an intercept-only model based on their contribution to the model’s explanatory power until no additional variables were significant. This approach helped identify the most influential explanatory variables affecting the response data. We also tested the effect of particular explanatory variables univariately on each of the response variables separately. The significance of each test was assessed by means of 999 Monte–Carlo permutations. The R-script is provided in the electronic supplementary material as an R-Markdown file.

The survey was distributed via email to stakeholder organizations, with a request to share it among their members, including hunters, nature conservationists, farmers and sheep and goat breeders. However, farmer organizations and sheep and goat breeder associations did not respond positively to this request. By contrast, hunter organizations and nature organizations did participate. The survey was also disseminated to the general public through social media platforms such as Twitter and Facebook. Further details on the survey’s dissemination can be found in the electronic supplementary material, table S1. Responses with blank or incomplete answers for key variables were excluded to ensure analytical consistency, following best practices in survey analysis [57,58]. Of the 1212 responses, 121 were removed owing to blank responses on more than one question (*n* = 1091). They were used for descriptive analyses. Another 20 responses with one blank question, were removed for the RDA, leaving 1071 surveys. With a sample size of 1071 respondents, 6.5 million Flemish people are represented with a 95% confidence level and a 3% margin of error; a sample of 393 Flemish hunters among an estimated 13 000 active Flemish hunters offers a 95% confidence level and a 5% margin of error. We do not claim this survey represents a random sample of opinions in Flemish society. Instead, the survey seeks to contrast a key stakeholder group (hunters) with the general public. Among stakeholder groups, there may be a non-response bias [57,58] since the theme of wolves probably solicits responses from individuals who are already outspoken about the subject. This may introduce skewness in the sampled perspectives.

## 3. Results

The respondents (*n* = 1071) represented roughly a mix of the general public and members of the stakeholder organizations. The average age of respondents was 43.06 years (s.d. = 13.87). The sample had a significantly higher proportion of males (*n* = 760; 71%) compared with the expected 50% (*Z* = 12.7, *p* < 0.0001). Among the respondents, 393 (37%) scored that they hunted or participated in hunting activities (considered as ‘hunters’) and 678 were non-hunters (63%). A total of 9.7% of hunters were female, versus 40.9% of females in the non-hunting group. Most of the respondents (86%, *n* = 921) did not reside in a city (*Z* = −23.71, *p* < 0.0001), and 66% (*n* = 707) had attained higher education (Bachelor’s, Master’s or PhD) (*Z* = 10.561, *p* < 0.0001). Nearly all respondents (89%, *n* = 953) reported spending time in nature daily or weekly (*Z* = 18.1, *p* < 0.0001). Many owned a dog (58%, *n* = 621*; Z* = −4.967, *p* < 0.0001), while fewer (37%, *n* = 396) owned a cat (*Z* = 8.68, *p* < 0.0001) or livestock (25%, *n* = 268*; Z* = 14.63, *p* < 0.0001).

**Figure 1.**
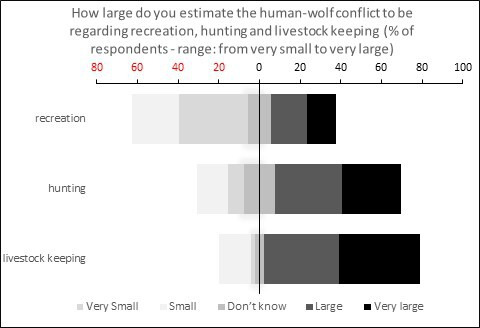
Estimated human-wolf conflict regarding recreation, hunting and livestock keeping according to the respondents

Among all respondents, 46% expressed a positive attitude towards hunting, while 40% held a negative attitude, with the remaining respondents being neutral. A slightly higher proportion (54%) had a positive attitude towards the return of wolves in Belgium, compared with 38% who viewed wolf presence negatively, with 7% remaining neutral. Regarding the origin of wolves, 54% of respondents were uncertain whether wolves had been reintroduced by humans or had returned spontaneously, while 37% believed in spontaneous return. Slightly more than half (54%) of the respondents believed there was room for wolves in Belgium, whereas 42% disagreed. The conflict between humans and wolves was perceived as large or very large by a majority of respondents concerning hunting (52%) and livestock keeping (77%), but concerning recreation most respondents (57%) thought the conflict was small or very small (figure 1). Additionally, 75% of respondents estimated there were five or fewer wolves in Belgium, while 25% believed there were more.

**Table 1.** RDA marginal effects per explanatory variable on attitudes and perception towards wolves for all respondents, only hunters and only non-hunters.

	all respondents		hunters		non-hunters	
variable	marginal effect *R*^2^	*p*‐value	marginal effect *R*^2^	*p*‐value	marginal effect *R*^2^	*p*‐value
attitude towards hunting	0.4702	0.001	0.120	0.001	0.271	0.001
hunter (0/1)	0.3313	0.001	NA	NA	NA	NA
dog ownership	0.0976	0.001	0.025	0.001	0.033	0.001
urbanization	0.081	0.001	0.022	0.002	0.034	0.001
time in nature	0.0423	0.001	0.011	0.009	0.027	0.001
gender	0.040	0.001	0.000	0.960	0.002	0.226
livestock ownership	0.033	0.001	0.015	0.009	0.043	0.001
education	0.020	0.001	0.034	0.002	0.006	0.587
cat ownership	0.014	0.001	0.015	0.458	0.001	0.715
living in wolf area	0.004	0.020	0.003	0.281	0.002	0.721
age	0.002	0.105	0.006	0.084	0.003	0.164

Analysis of the marginal effects of each explanatory variable in the overall dataset (table 1) reveals that the attitude towards hunting and being a hunter are very strongly correlated with the variables making up the ‘perspective’ on wolves. The final model of the forward selection analysis included six variables: attitude towards hunting, being a hunter, dog owner, livestock owner, age and being female. This model explained 50.07% of the total variance in the response data (*R*² = 0.5007, adjusted *R*² = 0.4978). However, only the attitude towards hunting showed a strong conditional effect. Even though statistically significant, the remaining variables combined explained less than 3% of the total variance. Table 2 shows the forward selection model results (adjusted *R*² values) on all respondents, only hunters and only non-hunters and the conditional contribution of additional variables. Only significant variables (*p* < 0.05) were included. All full models and FW selection models were highly significant (*p* < 0.001).

**Table 2.** Forward selection model results (adjusted *R*^2^ values) on all respondents, only hunters and only non-hunters and conditional contribution of additional variables.

variable	all respondents	hunters	non-hunters
full model	0.499	0.163	0.295
FW-selected model	0.499	0.163	0.294
hunting attitude	0.470	0.118	0.270
dog	0.011	0.016	0.006
hunting	0.007	NA	NA
livestock	0.004	0.009	0.011
age	0.004	0.007	0.004
gender	0.001	NS	0.004
education	0.001	0.010	NS

In univariate analyses comparing hunters and non-hunters, the following differences were observed regarding the five recorded components of attitudes and perceptions towards wolves (table 3). Hunters generally rated their attitude towards wolves as negative (78% negative, 10% neutral and 12% positive), in contrast to non-hunters (17% negative, 5% neutral and 78% positive). Whether a respondent was a hunter or not explained 43.4% of the variance in attitude towards wolves (*r*² = 0.434, *p* < 0.0001).

**Table 3.** Mean and s.d. of scores of hunters versus non-hunters for the five questions on attitudes and perceptions towards wolves, effect size (*r*^2^) and significance of the difference.

	attitude to wolves	wolf dispersal	belong in BE (Belgium)	conflict livestock	conflict recreation
mean hunters	−1.03	−0.65	−0.71	4.62	3.56
s.d.	1.11	0.70	0.68	0.63	1.24
mean non-hunters	1.12	0.62	0.59	3.61	1.95
s.d.	1.34	0.72	0.79	1.19	1.24
*r* ^2^	0.404	0.375	0.413	0.184	0.280
*p*	0.0001	0.0001	0.0001	0.0001	0.0001

**Figure 2.**
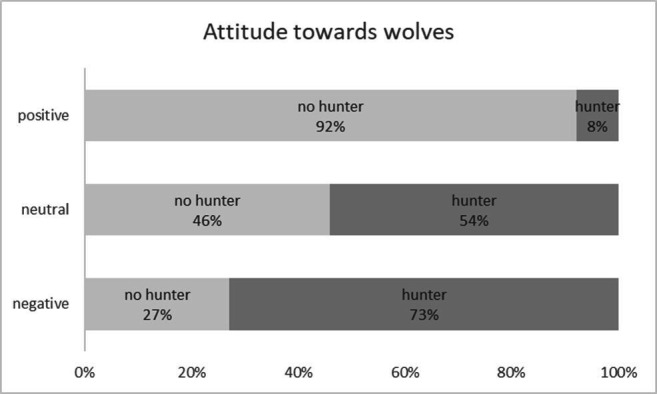
Attitude towards wolves by hunters and non-hunters.

**Figure 3.**
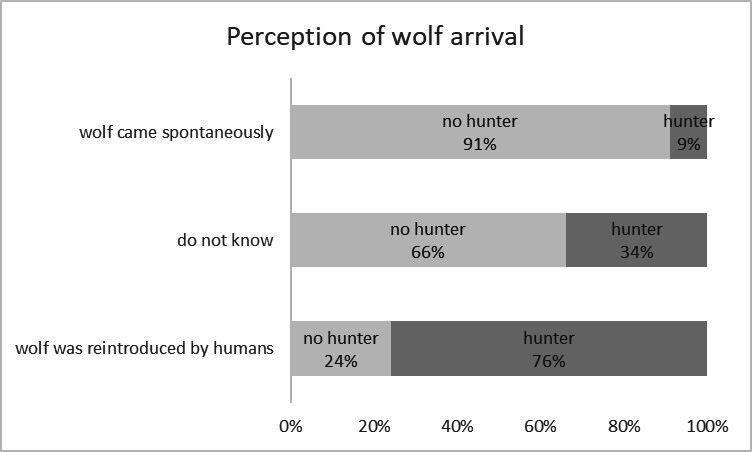
Perception of wolf arrival by hunters and non-hunters.

**Figure 4.**
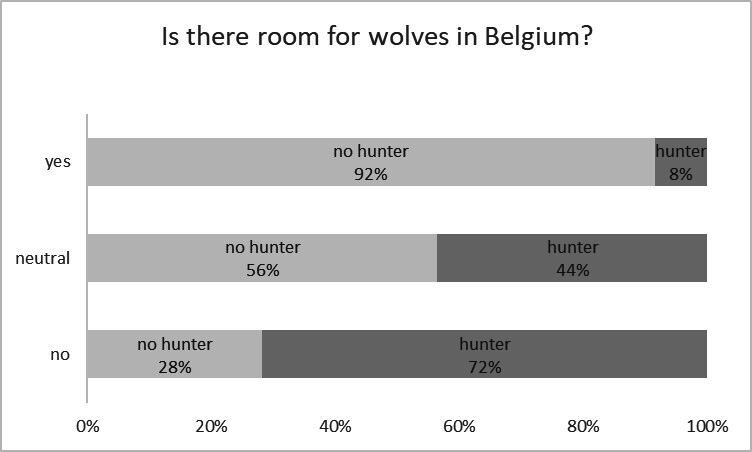
Perception of room for the wolf in Belgium, by hunters and non-hunters.

For other response variables, perceptions also typically diverged between hunters and non-hunters (table 3; figures 2–4). A significant majority of hunters (83%) believed there is no place for wolves in Belgium, compared with 19% of non-hunters. Additionally, 78% of hunters thought that wolves had been actively reintroduced and released, versus 14% of non-hunters. Moreover, among hunters, a higher percentage estimated the conflict with livestock ownership, recreation, and hunting to be large, compared with non-hunters.

**Figure 5.**
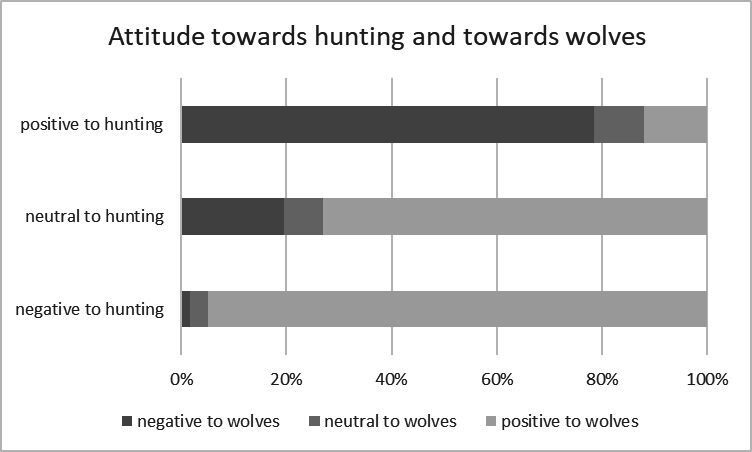
Attitude towards hunting and towards the presence of wolves in Belgium.

We performed multivariate linear regressions between data matrices on the total set of respondents (*n* = 1071), and on subsets containing only hunters (*n* = 393) or only non-hunters (*n* = 678). When regressing attitude towards hunting versus perspective on wolves, we see an even stronger effect (*R*² = 0.638, *p* < 0.0001). Attitude towards hunting thus explained more variation in attitude towards wolves than being a hunter, as already indicated by the forward selection RDA. Similar results were obtained when only considering hunters or only non-hunters. Even among hunters, the main variable explaining the response variables summarized as ‘perspective on wolves’, was attitude towards hunting, although the effect was weaker. Non-hunters who evaluated hunting positively tended to have a more negative attitude towards the presence of wolves in Belgium (cf. figure 5).

Likewise, among hunters, respondents who evaluated their fellow hunters more negatively tended to be more positive towards wolves. Even among hunters, the attitude towards hunting was the foremost variable, explaining more than 12% of the variation in response variables of the wolf perspective (cf. table 2).

After the inclusion of the respondent’s attitude to hunting, none of the other variables could explain more than 1% of the variance in perspective on wolves. Combined, these remaining variables accounted for only 3.3% of the variance in the wolf perspective, in contrast to 48.6% explained by attitudes towards hunting. This general pattern persisted when focusing exclusively on non-hunters or hunters.

## 4. Discussion

Our data provide insight into perspectives on wolves during the initial phase of wolf settlement in Flanders, with the aim to quantify the influence of several explanatory variables. This is particularly valuable owing to the specific timing of this context. People living in areas where wolves have been continuously present tend to hold more negative attitudes compared to areas where wolves have returned [10]. In our study, the respondents exhibited divided attitudes towards the return of wolves, with a slight majority (54%) expressing a positive outlook, whereas earlier surveys on attitudes towards wolves in other European countries reported that about 42% of the respondents had positive attitudes [12]. Flemish non-hunters are generally positive towards wolves, while hunters are generally negative, similar to other studies [10].

For the most part, we could confirm the significant impact of demographic and personal traits that are known to influence perspectives on wolves. Residency in a city or urban area, and to a lesser extent, a higher level of education, were associated with a positive view on wolves, consistent with other studies [3,16,50,59]. By contrast, residency in rural or wolf-populated areas, older age, lower levels of education, livestock ownership and particularly dog ownership, were linked to a negative perspective on the return of wolves, aligning with previous findings, including the higher importance of the first factor, i.e. rural or wolf-area residency [13,14,23,60]. In general, city dwellers face lower risks of conflict with large carnivores than rural residents [14,23,25]. In rural communities that coexist with wolves, supporters of wolf conservation are often perceived as disconnected urban elites imposing conservation agendas on rural land management, negatively affecting rural livelihoods and economies [61,62]. Regarding education levels, most studies report that higher degrees correlate with more positive wolf attitudes, while one-third of the studies find no association [10]. Another factor making people less tolerant of wolves is the owning of livestock, as these owners may identify with farmers or fear predation on their animals [22]. An influence of age is not seen in all wolf surveys, but if a correlation is present, usually older respondents hold more negative attitudes [10], as in our study, confirming intergenerational differences in perspectives towards wolves. Cat owners showed a higher percentage of positive perspectives compared to non-cat owners, a finding that needs to be confirmed by further research. It was found that people identifying as ‘cat-people’ score lower on dominance-related traits than ‘dog-people’ [63]. Speculatively, if these personality traits relate to an absence of a domination worldview, it could explain a more positive attitude towards wolves [20]. We also found that individuals who spent little time in nature, typically had more negative attitudes, in contrast to Roskaft *et al*'s findings [13]. However, our survey question focused on ’spending time in nature’, which may have been interpreted differently from ’engaging in nature activities’, as phrased in the other study; the latter may resonate more strongly with individuals who are particularly nature-oriented. We found significantly more female than male respondents with a positive attitude towards wolves, although the effect of gender was limited. It is known that gender effects tend to vary among studies [16,17,23]. Interactions between factors are possible. In our study, the factor of gender may have been entangled with other aspects, as a relatively higher proportion of female respondents resided in urban areas, and in general, women tend to be less involved in hunting [64].

The multivariate analysis identified six key variables that were most influential in explaining the response data: attitude towards hunting, being a hunter, dog ownership, livestock ownership, age, and being female. The final model incorporating these six variables explains about 50% of the variation in the response data. While the significance of these explanatory variables in shaping perspectives on wolves is not new, their marginal effect sizes (*R*²) were small. This contrasts sharply with the two most significant explanatory factors—engagement in hunting activities and especially attitude towards hunting—which play a crucial role in shaping responses. It is interesting to study how the effects of other explanatory variables with relatively weak explanatory strength, may be mediated by these strong explanatory factors, i.e. involvement in hunting and attitudes towards hunting. Being a hunter and the degree to which respondents endorse hunting strongly covary with their perspective on wolves, revealing a negative symmetry: non-hunters are generally positive towards wolves, while hunters are generally negative. The nearly uniform rejection of wolves by hunters is striking and aligns with findings from other European countries (The Netherlands: [20]; Spain: [31,65]; Norway: [23]). Hunters in Finland explain their negative views by citing three main reasons: wolves damage human livelihoods, injure and kill hunting dogs, and pose a perceived threat to safety [36]. Our data show that relatively more hunters (69%) than non-hunters (57%) perceive a conflict between the presence of wolves and hunting, which is consistent with other studies (e.g. [12]). This conflict may be direct, owing to protective measures that limit hunting in core wolf territories, or indirect, through competition for prey species targeted by hunters [2,12]. In areas where wolves have returned, conservation management plans often include restricting traditional hunting practices, which is perceived as reducing the quality of life for local hunters [38]. In Latvia, negative attitudes among hunters were associated with beliefs that there are too many wolves, that wolves are dangerous, and that they cause damage to farmers and financial losses. A more negative attitude was linked to a willingness among respondents to decrease wolf populations. Hunters were more likely to have a positive attitude towards wolves as long as they could hunt them [66]. In Norway, wolf presence was found to reduce landowners’ revenues from small game hunting and cause greater economic variability in rural communities [67]. Similarly, in Belgium, financial considerations may significantly influence attitudes towards wolves, as renting hunting grounds, particularly for larger game species, can be very costly. Nevertheless, some studies pointed out that some hunters value the presence of wolves [11,37]. Interestingly enough, views on hunting activities seemed to be a strong explanatory variable within both hunter and non-hunter groups. More specifically, hunters with negative views on hunting exhibited more positive perspectives on wolves, whereas non-hunters with positive views on hunting showed more negative perspectives. The reasons why some hunters hold negative attitudes towards hunting, and how these hunters differ from their peers, are unclear. This suggests that hunters are not a homogenous group and that they may have diverse motivations for hunting and differing ethics. Some hunters have negative views on certain prevailing hunting practices and make a distinction between good and bad hunting practices. This prompts the need for further research and conversations about hunting ethics [68].

Our findings align with previous research that emphasises the role of values and attitudes in shaping perspectives on wildlife and conservation. Earlier studies suggest that attitudes towards large carnivores are linked to broader perspectives on the human–nature relationship, which is an important aspect for future research. Individuals who see the human role as ‘stewards of nature’ tend to have the most positive perceptions of large carnivores. Conversely, a ‘master over nature’ perspective, which was observed among Belgian hunters, tends to predict a negative perception of wolves [20]. Debates about wolf conservation may be less about the wolves themselves and more about conflicts between groups with opposing views about the value of wildlife, land-use rights and traditional ideas about wildlife use versus conservation [61]. Wildlife value orientation is a cognitive concept developed to study public beliefs about wildlife [69]. Mutualism, characterized by social affiliation and caring beliefs, and domination, characterized by beliefs about appropriate use and hunting, were identified as key drivers of attitudes towards wildlife [70,71]. A sociocultural index that quantifies domination or mutualistic views could help provide a deeper understanding of public relationships with wildlife [72].

Our results suggest that the rejection of wolves is not based on factual knowledge. A majority of hunters believed that wolves had been actively reintroduced and released, despite evidence showing that wolves naturally recolonized the area. This misconception has long been prevalent within the hunting community and is not unique to Belgium; it has also been documented in other parts of Europe where wolves have recently recolonized areas they previously had to abandon [73]. The narrative of ‘introduction by humans’ may reflect underlying power dynamics, where certain groups perceive science-based wolf conservation as a threat to their economic and cultural interests [38]. Similarly, a large majority of hunters believed there was no room for wolves in Belgium, while most non-hunters held the opposite view. The question of whether there is space for wolves can be examined from both ecological and societal perspectives. Ecologically, there is sufficient habitat within Belgium to support at least 10 to 20 wolf packs [48]. However, the societal perspective is more subjective. To foster a broader appreciation for the ecological value of wolves in the ecosystem and to dispel myths and misinformation, hunters could be involved in conservation efforts and educational programmes.

The significance of dog ownership in shaping attitudes towards wolves is noteworthy, particularly given the educational potential for informing the public about risks and promoting good practices as modes of risk mitigation. Wolves have been reported to kill dogs, including livestock guarding dogs, especially when the wolves outnumber the dogs [7,26,69,74–76]. Hunting dogs are frequently attacked by wolves in Finland and other parts of Scandinavia [24,77]. Generally, individuals who have lost a pet to wolves tend to have more negative attitudes towards them [25]. Hunting dogs are most at risk of being attacked near wolf territory boundaries, suggesting that wolves primarily view dogs as competitors [34]. In some regions, intraspecific aggression towards unfamiliar wolves that invade territories is the leading cause of wolf mortality [78]. However, wolves have also been observed to display an ‘aversion to fighting’ and to avoid aggressive encounters with dogs [79]. The fear of losing valuable dogs to wolves can significantly impact the lifestyle of rural residents [67]. This probably explains the significant effect of dog ownership on attitudes towards wolves in our study, second only to ‘attitude towards hunting’. Similar to the general public, hunters also fear potential damage to their dogs caused by wolves. For some hunters, the bond and cooperation with their dog are more rewarding than the act of hunting itself [38]. In Croatia, dogs are often killed during drive hunts for boar, with many being consumed by wolves [33]. Given these risks, researching, formulating, and disseminating best practices among hunters and non-hunter dog owners could be highly beneficial. In our survey, we did not distinguish among pet dogs, guardian dogs, and hunting dogs in relation to the observed attitudes, which could be an interesting factor to assess in future studies.

In our study, higher estimations of conflict were interpreted as indicative of a more negative perspective on wolves, while lower estimations were seen as more positive. The respondents’ perception of high conflicts between livestock keeping and wolves, and hunting and wolves corresponds to previous studies [10]. Although plausible, our interpretation might not fully capture nuanced views. For example, individuals perceiving a high level of wolf-livestock conflict could still hold a generally positive attitude towards wolves but be well-informed about livestock depredation incidents. As the accuracy of respondents' perceptions aligns with reality, disentangling general attitudes towards wolves from specific knowledge about human-wolf interactions becomes more complex.

As attitudes can be dynamic, and few studies support a long-term ‘happy coexistence’, it would be interesting to repeat the study to see how this will develop after a longer time with wolves being present [10]. Public opinions on human-wolf coexistence typically illustrate a ‘wicked problem’, characterized by divided opinions and the absence of clear solutions owing to the divergent values of stakeholders and complexities of the socio-ecological environments in which the issue is embedded [80]. Therefore stakeholder participation is of key relevance in these studies. While several hunter and nature organizations complied with the request to distribute the survey, the farmers' organization did not. Stakeholders may perceive science as unbeneficial and therefore may be more likely to distrust scientific information on the issue [81]. Such distrust could explain the farmers' organization’s refusal to participate in a scientific project on wolves. Given that hunters may also harbour similar scepticism [81,82], their high willingness to respond was particularly valuable. Hunters are important stakeholders in natural and rural landscapes, with a major influence on the conservation success of large carnivores. Increasing efforts to engage hunters in dialogue and to include hunters in research projects could help bridge the perceived gap between scientists and hunters, as well as other groups such as farmers. Collaboration between environmentalists and hunters in nature conservation and land stewardship could benefit mutual interests, including tourism and ecosystem services [20,83]. When attempting to foster understanding, it should be considered that access to information about wolves can sometimes polarize attitudes further, rather than harmonize them [84]. Social media exposure to opposing views can either increase [85] or decrease [86] political polarization, highlighting the need to explore both emotional dispositions and cognitive perspectives related to wildlife [87,88]. Communication strategies should involve in-group messengers and focus on shared values, such as the welfare effects of nature and individual freedoms [89]. Hunter opposition may not only be about the impacts of wolves but also about defending their social identity and perceived freedoms against other interest groups. Addressing these group dynamics can advance conservation and conflict management [90]. Social dynamics and confirmation bias, where individuals consume information that reinforces pre-existing beliefs [91,92], should be considered. In Flanders, the establishment of a territory by a GPS-collared wolf was used by some to argue that wolves had been secretly released, illustrating how facts can be used to reinforce existing biases. Conservation managers should be aware of my-side bias—favouring one’s own viewpoint—and explore de-biasing mechanisms [93]. The important influence of values on attitudes is not restricted to wolf conservation (e.g.: forest conservation [94]; environmental concern [95]). Recognizing the diverse value systems of all involved in the stakeholder-ecosystem relating to wolf conservation (including scientists), with respectful engagement, co-creation, and mutual understanding is crucial for effective management [96]. Adopting stakeholder-focussed value-based approaches can lead to more sustainable and effective conservation strategies [80,97].

## 5. Conclusion

After the return of the wolf in Flanders, people’s perspectives on wolves were divided. This was influenced by several explanatory socio-demographic factors, but above all, there was a strong link between perspectives on wolves and attitudes towards hunting. It is important to further examine to what extent attitudes towards hunting could serve as a proxy for perspectives on wolves and how they mediate the other variables under study. Studies that seek to validate the measurement of attitudes towards hunting or ‘hunting beliefs’ could provide valuable insights [98], as well as studies quantifying domination or mutualistic views [72]. Acknowledging the diversity of perspectives, and the importance of deeper values and ethics as drivers is crucial. Applying social science insights may help to improve stakeholder involvement and can contribute to effective and respectful wolf conservation management.

## Data Availability

The dataset is available as an Excel-file in the electronic supplementary material, as well as other supplementary information (e.g. translated survey, map, …). The Rmd script to reproduce the analysis is also available online [99].
